# Supplementary material for: Multifunctional Cell Regulation Activities of the Mussel Lectin SeviL: Induction of Macrophage Polarization toward the M1 Functional Phenotype
Source: Mar Drugs. 2024 Jun 11;22(6):269. doi: 10.3390/md22060269 (PMC11204705; doi:10.3390/md22060269)
Supplement: Supplementary file 1 [file marinedrugs-22-00269-s001.zip › marinedrugs-3002543-supplementary.pdf]

Supplemental data

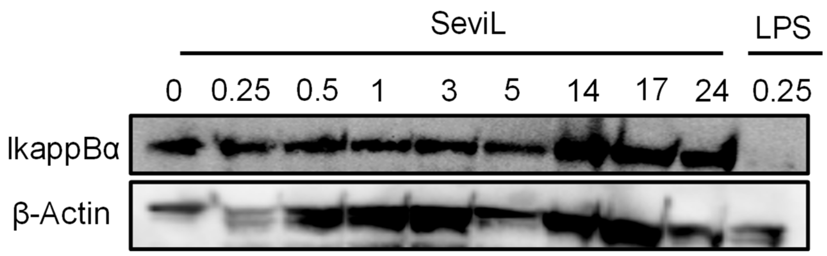

Figure S1: Effect of SeviL and LPS on IkappaBα in RAW264.7 cells.

SeviL (10 μg/ml) or LPS (1 μg/mL) were treated for several time points. The number from 0 to 0.25 indicates the treatment time (hour). Appearance of IkappaBα negatively regulates the activation of NFκB.
